# Unusual presentation of tumor‐induced osteomalacia mismanaged due to misdiagnosis: A literature review based on a case report

**DOI:** 10.1002/ccr3.8885

**Published:** 2024-05-20

**Authors:** Mahdieh Fatollahzadeh, Hamid Reza Aghaei Meybodi, Hamid Pajavand, Moloud Payab, Mahbube Ebrahimpur, Pouya Ebrahimi

**Affiliations:** ^1^ Endocrinology and Metabolism Research Center Endocrinology and Metabolism Clinical Sciences Institute, Tehran University of Medical Sciences Tehran Iran; ^2^ Evidence Based Medicine Research Center Endocrinology and Metabolism Clinical Sciences Institute, Tehran University of Medical Sciences Tehran Iran; ^3^ Non‐Communicable Diseases Research Center Endocrinology and Metabolism Population Sciences Institute, Tehran University of Medical Sciences Tehran Iran; ^4^ Elderly Health Research Center Endocrinology and Metabolism Population Sciences Institute, Tehran University of Medical Sciences; ^5^ Tehran Heart Center Cardiovascular Disease Research Institute, Tehran University of Medical Sciences Tehran Iran

**Keywords:** bone tumor, fracture, musculoskeletal, oncology, phosphate, tumor‐induced Osteomalacia

## Abstract

**Key Clinical Message:**

Tumor‐induced osteomalacia is a rare but potentially serious disease with nonspecific misguiding manifestations that can result in a wrong diagnosis and being treated for rheumatologic or other similar diseases. In patients with unexpected fractures, resistant musculoskeletal pains, and hypophosphatemia, this diagnosis should be considered by the physicians and approached through a complete history taking, physical exam laboratory, and radiologic evaluation to give the opportunity of on‐time treatment to the patient.

**Abstract:**

Tumor‐induced osteomalacia (TIO) is an uncommon mesenchymal tumor that results in disproportionate phosphorus excretion, primarily leading to bone‐related symptoms. Laboratory, imaging, and histopathological evaluation can confirm this pathologic condition. In this case, we present the history and subsequent clinical parts of a 50‐year‐old woman who presented with an unusual presentation of generalized musculoskeletal pains and a right ankle mass. Her disease was diagnosed with multidisciplinary evaluation and was approached by a surgical treatment. The patient was treated with total resection of the tumor, which led to complete resolution of musculoskeletal and metabolic abnormalities, which were resolved following total tumor resection. TIO is a paraneoplastic disease that results in abnormal secretion of phosphatonins, particularly fibroblast growth factor 23 (FGF23). This can cause hypophosphatemia, hyperparathyroidism, lower bone density, and increased risk of pathologic fractures. These tumors are mostly cured by surgical ± radiotherapy. The present study aims to provide insight into the fact that a TIO diagnosis is not always straightforward. However, in suspicious cases such as unexplained hypophosphatemia, it should be considered to prevent delayed diagnosis of the progressive pathology. The earlier treatment can prevent several complications and reduce the risk of mortality.

## 1 INTRODUCTION

Osteomalacia is characterized by insufficiency or delay in the bone mineralization process in mature bones, which can finally result in lower bone density and pathologic fracture.^1^ There are several reasons for this condition, ranging from usual bone mineral density (BMD) decline due to the process of aging or vitamin D deficiency caused by gastrointestinal disease (declined Vitamin D absorption), renal diseases (increased phosphate loss or insufficient processing of vitamin D), inadequate food intake, or lack of appropriate exposure to sun as well as genetic disorders (including autosomal dominant and X‐linked hypophosphatemia rickets).^2^ One of the least commonly seen causes of osteomalacia is caused by parenchymal tissue tumors, namely tumor‐induced osteomalacia (TIO).^3^

TIO, a rare paraneoplastic syndrome, can hinder renal phosphate reabsorption and cause hypophosphatemia through the secretion of fibroblast growth factor 23 (FGF23).^4^, ^5^ As a phosphaturic hormone, FGF‐23 inactivates 1, 25‐dihydroxyvitamin D, leading to compensatory hyperparathyroidism. TIO syndrome can be associated with manifestations such as muscle and bone pain and recurrent fractures that may lead to height loss.^3^ Diagnosing this disease is challenging due to the nonspecific symptoms and sometimes the co‐existence of other conditions.^4^ Therefore, it may remain undiagnosed for a long time.^5^

This study presents a rarely reported but educationally crucial case of TIO that had been under medical observation and treatment with the provisional diagnosis of rheumatologic diagnoses over several years and ultimately presented to our attention with bone pain and an inability to walk. This study aims to improve clinicians' and researchers' knowledge and understanding of the diagnosis of TIO in patients presenting with nonspecific clinical musculoskeletal and laboratory signs and symptoms.

## 2 CASE PRESENTATION

### 2.1 Case history and examination

A 50‐year‐old woman was admitted to our hospital because of generalized bone pain and a right ankle mass. 4 years before this admission, the patient had mechanical joint pain without inflammatory symptoms. She was originally diagnosed with seronegative spondyloarthropathy and received treatment for a prolonged duration involving corticosteroids and methotrexate. The patient's symptoms were under limited control. However, subsequent investigations revealed that she had hypophosphatemia. In her physical exam, there were no remarkable abnormalities except for a bony mass identified in the right ankle area. The patient stated that this tumor had gradually grown over time.

### 2.2 Methods

**FIGURE 1:**
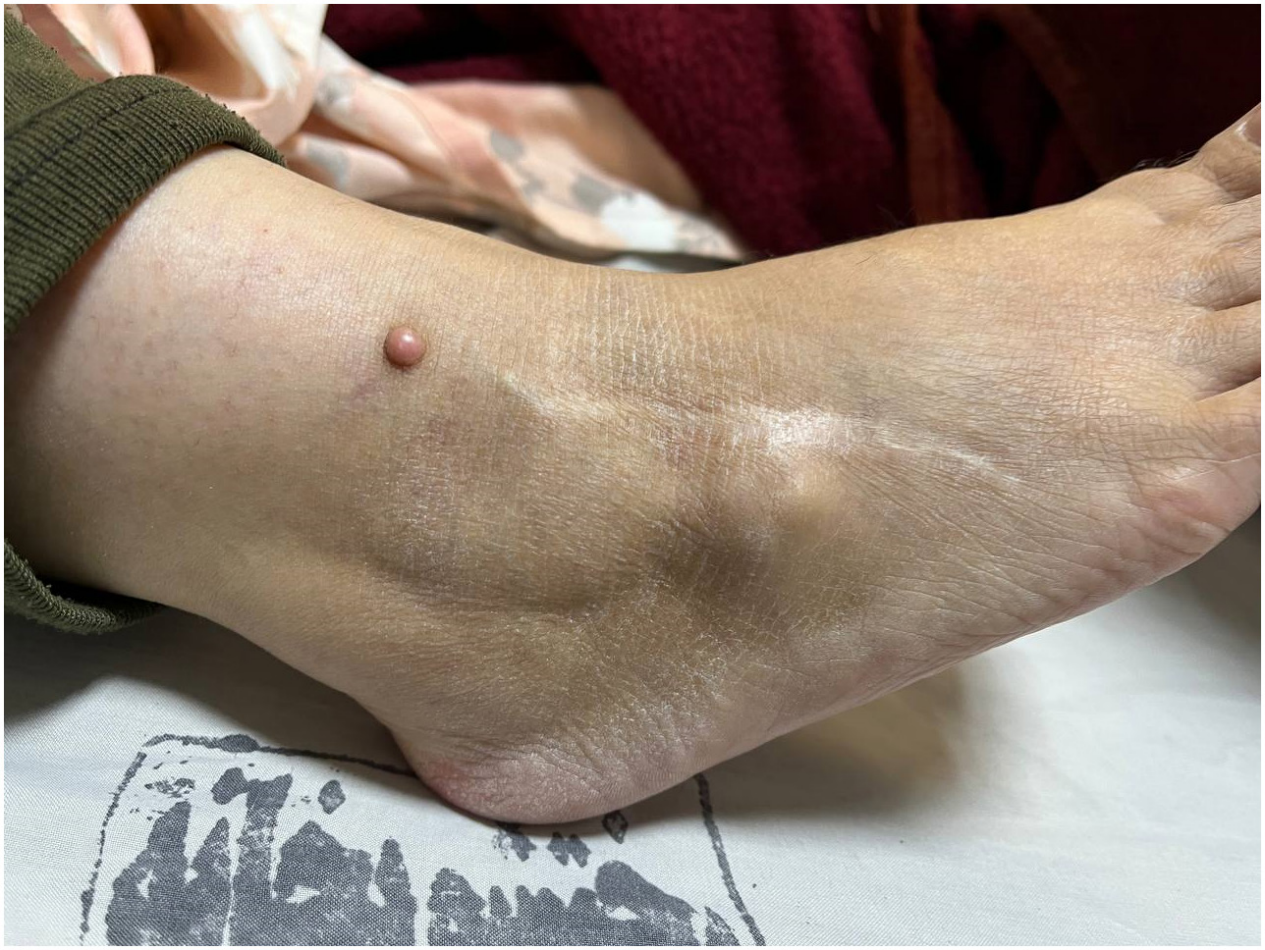
A bony mass was identified in the lateral part of the right ankle above the lateral malleolus.

**FIGURE 2:**
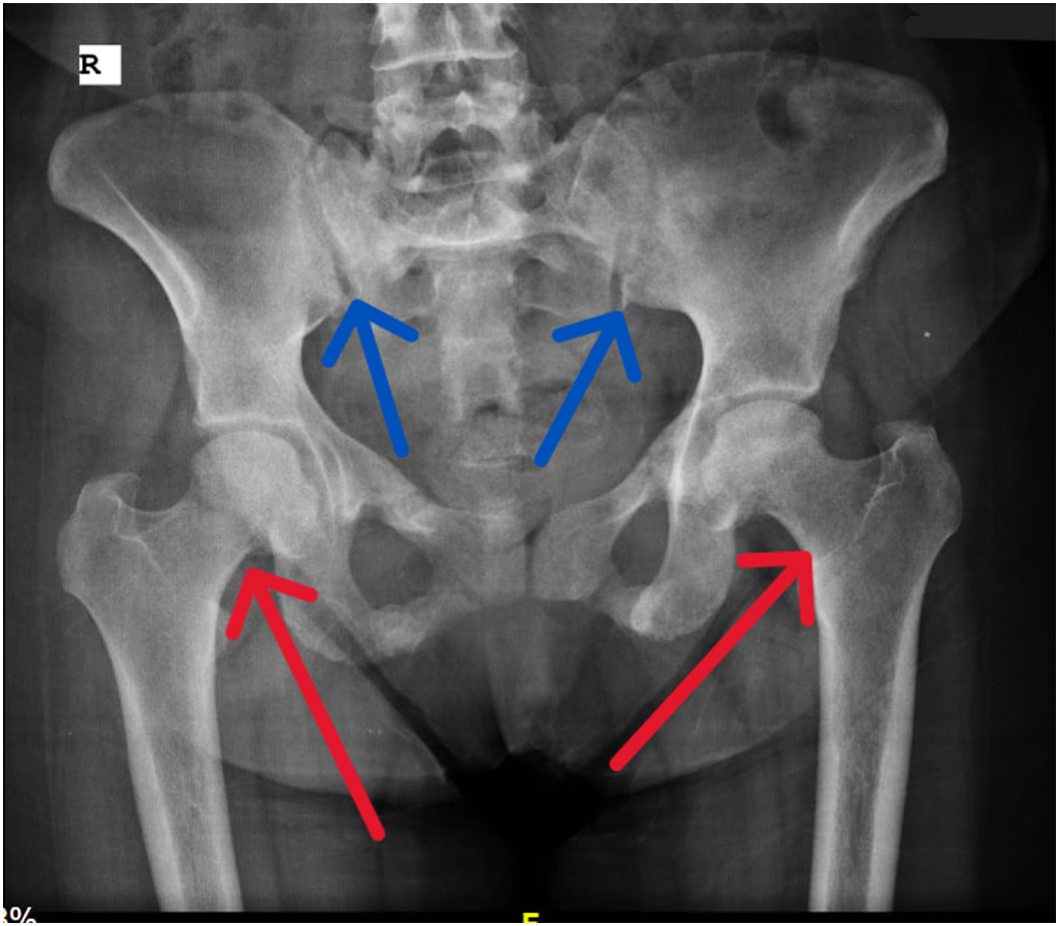
A radiograph pelvic plain X‐ray showed radiolucency in the bilateral femoral neck (Red arrows) and pelvic (blue arrows), suspicious of low bone mineral density (osteoporosis or osteopenia).

To investigate the nature of the tumor, a Ga DOTATATE PET/CT scan was performed, and the level of FGF 23 was measured. Consequently, by diagnosis of TIO, she underwent surgery to remove the mass. Surgical pathology revealed a tumoral tissue composed of spindle cells with small nuclei in favor of a mesenchymal phosphaturic tumor. After the surgery, her serum phosphorus level mildly improved, and she experienced relief from symptoms. In the one‐year follow‐up, Joule's solution was prescribed due to the gradual decline in serum phosphorus levels. The patient did not continue with the follow‐up and returned 3 years later, complaining of lameness as a result of severe hip pain and generalized bone pain. On examination, a recurrence of the tumor was observed at the site of the prior surgery (Figure 1). The tumor manifested as a noticeable bony lesion in the 5th metatarsus, which had originated a year prior and progressively enlarged. Laboratory findings were as follows: hypophosphatemia (1.5 mg/dL, reference range 2.6–4.5), elevated serum alkaline phosphatase (309 U/L, reference range 80–300), inappropriately high urine phosphorus (991 mg/day, reference range 340–1000), normal serum calcium (8.8 mg/dL, reference range 8.5–10.5), normal serum 25(OH)D (32 ng/mL, reference range 30–100) and elevated serum parathyroid hormone (104 pg/mL, reference range 11–67). Sex hormones, including FSH and LH levels, were within normal limits. Nevertheless, the concentration of FGF‐23 was 43 pg/mL (reference range 18–108). Due to pelvic pain, a pelvic X‐ray was obtained, which showed bilateral femoral neck and pelvic radiolucency (suggestive of low bone density), suspicious for the diagnosis of osteoporosis or osteopenia (Figure 2). BMD was measured and revealed a lumbar spine T‐score of 1, a femoral neck T‐score of −1.5, and a total hip T‐score of −1.6, confirming the diagnosis of osteopenia. An MRI was also performed, and it was found that the patient had bilateral hip avascular necrosis (AVN). Therefore, the diagnosis of recurrence of tumor secreting phosphatonins causing hypophosphatemia was raised due to recurrence of symptoms, low phosphorus, and growing mass at the previous surgical site. Owing to the extensive tumoral involvement, a treatment plan was proposed that included amputation at a higher level or radical surgery and removal of surrounding tissues. The decision was discussed with the patient and recommended, and alternative options were illustrated, ensuring she had enough information to decide. The patient refused to get amputated, so a radical surgery was performed.

### 2.3 Result and follow‐up

The abnormalities in biochemical markers returned to normal, and clinical symptoms resolved within a few weeks after surgery. She has been referred for orthopedic follow‐up for hip AVN fixation. Pathology examination revealed a mesenchymal phosphaturic neoplasm with osteoblastoma‐like morphology. Immunohistochemical staining indicated that the tumor was CD 99 (+), ERG (+), STATB2 (+), and Ki67 (low proliferative activity). The patient's symptoms have been alleviated remarkably, and she had no complaints of new or worsening symptoms. All procedures in this study were conducted by the ethical standards of the institutional research committee(s) and the Declaration of Helsinki (revised 2013).

## 3 DISCUSSION

This report describes a 50‐year‐old woman with mesenchymal TIO, a rare hypophosphatemia syndrome. Laboratory and histopathological data confirmed the diagnosis, and the metabolic abnormalities resolved after complete tumor resection. TIO produces paraneoplastic syndrome by secretion of phosphatonins, in particular, FGF23.^6^, ^7^ FGF23 causes hypophosphatemia through renal phosphate wasting, and a decline in 1, 25‐dihydroxy vitamin D synthesis leads to osteomalacia, which is characterized by muscle weakness, diffuse bone pain, and multiple fractures.^8^ Most tumors originate from the body's mesenchymal tissue.^9^ In 1991, Weidner et al. classified TIO into four categories based on histopathological features: phosphaturic mesenchymal tumor mixed connective tissue variant (PMTMCT), osteoblastoma‐like variant, ossified fibroids‐like variant, and non‐ossifying fibroma‐like variant.^10^

Despite advancements in understanding the disease, diagnosing and treating TIO remains challenging due to the variable size and distribution of the causative tumor in the body, which even advanced imaging techniques may fail to detect.^11^ Ariadne Bosman reported that the tumor was found on physical examination in 32.4% of cases. Despite local symptoms such as redness, swelling, and pain, in only 9.3% of cases, the tumor could not be seen during physical examination. Secondary symptoms such as weakness or proximal muscle pain were also present in 89.9% of cases, and the prevalence of tumors in the upper and lower limbs (91.9% and 91.3%, respectively) was higher than in the trunk and pelvis (82.8% and 85.9%, respectively).^8^

TIO, by definition, is a deficiency in bone mineralization. Bone fragility can be a diagnostic feature of the disease, as shown by the high rate of fractures in people with TIO (82%).^12^, ^13^ This study investigated the effect of TIO on bone density and found that the patient has low bone mass. In addition to laboratory and histopathological results, we used radiographic and MRI methods to diagnose TIO by indicating the tumor's location and its generated fractures. While other studies have used tools like F‐18FDG PET/CT, which is a highly sensitive but nonspecific diagnostic tool, to diagnose such a patient.^14^, ^15^ On the other hand, 68Ga DOTATATE PET is a useful diagnostic tool for localizing TIO tumors.^16^ Haeusler et al. suggested measuring FGF‐23 levels is necessary to diagnose tumors. However, serum level of FGF‐23 within the reference range cannot rule out the diagnosis of TIO.^17^ In our study, the FGF‐23 levels were within the normal range, possibly due to other phosphatonins.

In most cases, TIO is treated with surgery. In cases where surgery is not feasible or localization of the tumor is not possible, lifelong medical therapy with oral phosphate and calcitriol is required. Other treatments may be used, such as biological antibodies that target FGF‐23.^18^ In addition, radiotherapy may be used as an adjuvant treatment for tumors that cannot be completely removed.^19^

TIO is an uncommon paraneoplastic syndrome that arises from the secretion of FGF‐23 by small mesenchymal tumors, leading to hypophosphatemia, vitamin D deficiency, and osteomalacia. Diagnosis is delayed due to the slow progression of symptoms. Surgery is the primary and most efficacious approach, and alternative modalities such as radiotherapy may be employed in cases where complete tumor removal is not feasible.

## 4 CONCLUSION

TIO is a rarely seen and reported but potentially serious medical condition with a broad range of nonspecific and misguiding manifestations that can result in a wrong diagnosis. It can lead to being inappropriately treated for rheumatologic disease or other misdiagnosed bone pathologies **(**Table 1 **).** In patients with abnormal fractures, resistant musculoskeletal pains, and hypophosphatemia, this diagnosis should be considered by the physicians and approached through a complete history taking, physical exam laboratory, and radiologic evaluation to give the opportunity of on‐time treatment to the patient. Most cases are cured with surgical resection of the parenchymal cells, but sometimes supplementary radiotherapy or more invasive therapeutic decisions are required to be.

**TABLE 1:** Tumor‐induced osteomalacia literature review.

Article and authors	Age and Gender	Presentation	Treatment/Progression or remission of the disease
Dadoniene. Et al. 2015^20^	48‐year‐old male	Hx: Gradually worsening weakness, musculoskeletal pain, declined height and weight, + history of cancer in both parents Ph/Ex: Gradually worsening scoliosis and kyphosis, weight loss, overall weakness, muscle weakness, and pain by touch RAD: Compression Fx in T11 and T12 vertebrae confirmed by X‐ray‐CT‐MRI, declined BMD, New Fx in T7‐T8, WBBC: Increased Uptake in multiple bones, More spine Fx, and irreversible malformations Lab: Declined Ph, Increased Alkp, Increased FGF3, NL Ca & VitD	Tx: Initially with NSAID; No response. Change the Tx to bisphosphonates: No response; after several years: true Dx: surgical resection of the Mesenchymal tumor Histopathology: Confirmed TIO Prog: Partial resolution of signs and symptoms, Irreversible malformations remained, Improved Alkp, Ph, FGF3 level
Kobayashi. et al. 2011^21^	53‐year‐old female	Hx: Progressive bone pain and weakness for several years, declined height, Vx bone Fx, Hip Fx Ph/Ex: Gradually worsening weight loss, overall and muscle weakness RAD: Compression Fx in Vx. WBBC: multiple Increased Uptake areas more intense in the head and neck (right), More spine Fx, and irreversible malformations Lab: Declined Ph, Increased Alkp, remarkably increased FGF23, impaired Ph reabsorption in kidneys	Dx was made 4 years after the initiation of symptoms, with a whole body scan and MRI Tx: Angiographic embolization; partial response, Skull base resection Histopathology: Phosphaturic mesenchymal tumors (TIO), FGF‐23 secreting tumor Prog: Complete resolution of resolution of clinical integrity, Improved Alkp, Ph, FGF3 level. No recurrence in 13‐month FU
Romualdo‐Silva. et al. (2009)^22^	45‐year‐old male	Hx: knee, ankle & feet Pain progressed to all over the body for 4 years w/o dx; symptoms progressed to severe muscle pain Ph/Ex: severe overall and muscle weakness, worsening Thoracic Kyphosis, after 6 years, a 3 cm round mass at lateral thigh RAD: Several Compression Fx in Vx, hip, femur, and thorax, declined mineralization. WBBC: Multiple increased Uptake areas, more intense in the head and neck (right), More spine Fx, and irreversible malformations. Lab: Declined Ph, Increased Alkp, remarkably increased FGF23, declined 1.25. OH, VitD	W/O dx initiated Tx with Ca, Ph, Calcitriol Dx, which improved his condition significantly Tx: resection of tumor, pathologic confirmation Histopathology: Phosphaturic mesenchymal tumors, mixed connective tissue type (PMTMCT) Prog: Complete resolution of resolution of clinical integrity, Improved lab data, No recurrence in 12‐month FU
Li. Et al. 2022^23^	NR Year‐old male	Hx: Progressive bone pain and decline in height Ph/Ex: acromegalic physical features RAD: MRI; pituitary somatotroph adenoma, Octreoscan: Right femur tumor Lab: elevated GH and IGF‐1 Declined Ph, Increased impaired pH reabsorption from kidneys	Tx: First resection of foot bone tumor and then resection of pituitary adenoma. Remarkable improvement in his/her condition Prog: FU
Arai. Et al.^24^	39‐year‐old male	Hx: Low back pain chest, right hip, and bilateral foot pain. One year after pain initiation, enlarging elastic soft masses in the plantar side of the right hallux Ph/Ex: Gradually worsening weight loss, overall and muscle weakness RAD: cystic radiolucency in Rt 4th metatarsal bone, Lt 3rd and 4th metatarsal bone, and in Rt pubis, WBBC: multiple Increased Uptake areas Bilat rib, right pubis, Bilat tarsus, Rt 4th metatarsal bone, Lt 3rd and 4th metatarsal bone. MRI: 3 soft tissue mass in right flexor hallucis longus Lab: Declined Ph, Increased Alkp, remarkably increased FGF23 more in Rt common iliac vein and Rt ext femoral, impaired Ph reabsorption from Kidneys	Tx: Removal of all soft tissue mass Histopathology: Confirming the TIO Dx Prog: all lab data became NL, and clinical symptoms improved and disappeared in 2‐month FU
Zhang. Et al. (2023)^25^	36‐year‐old female	Hx: progressive systemic skeletal AbNL, LBP during the last 2 years Ph/Ex: multiple thoracic Vx tender & pain with palpation, Curved thoracic spine RAD: Several Compression Fx in Vx, Rt Thoracic Vx curve, declined mineralization. WBBC: multiple Increased Uptake areas more intense in the head of Rt femur➔ shown in PET scan and Increased BM metabolism, multiple Fx Lab: Declined Ph, Increased Alkp, remarkably increased FGF23, declined 1.25. OH, VitD, Increased PTH	W/O dx initiated Tx with Ca, Ph, and Calcitriol, but no response was seen Tx: CT guided resection of tumor, simultaneous Pathologic confirmation of right femoral and bone grafting Histopathology: Phosphaturic mesenchymal tumors, positive for CD56, SATB2 and FLI‐1 Prog: Complete resolution of laboratory, clinical, and radiologic AbNL and full recovery
Kumar. et al. (2022)^26^	30‐year‐old male	Hx: 4‐year Hx of severe muscle aches, bone pain, and proximal muscle weakness, Ph/Ex: tenderness to palpitation, decreased muscle force, RAD: localized Rt femoral mass, increased bone uptake Lab: Declined Ph, Increased Alkp, remarkably increased FGF23, urinary phosphate wasting Histopathology: Mesenchymal tissue (TIO)	Tx: Radiofrequency and pharmacotherapy improved all lab data. Clinical condition improved remarkably. Histopathology: Phosphaturic mesenchymal tumors (TIO), FGF‐23 secreting tumor, Prog: Complete resolution of resolution of clinical condition, Improved Alkp, Ph, FGF3 level. No recurrence in FU
Aligail.et al. (2022)	78‐year‐old male	Hx: referred to the endocrine clinic due to generalized severe OP dx by DEXA, generalized body pain, and muscle weakness for 7 years. Ph/Ex: Significant proximal hip & shoulder weakness, muscle wasting, bed bound, declined height, kyphosis, bilateral chest crackle RAD: PET increased uptake in the soft tissue of the posterior aspect of the Rt humeral head, MRI: ill‐defined nodular lesion corresponding with the initial area of uptake, measuring 12 × 12 mm, related to the distal teres minor muscle fibers, Multiple Fx in Femur, ribs, Lab: Declined Ph, Ca & 25(OH) VitD, Increased Alkp, PTH is inclined, significant increase in FGF23, urinary phosphate wasting Histopathology: Mesenchymal tissue (TIO)	Tx: medical Tx with oral Ph, Ca, no clinical improvement, and no change in Ph, FGF 23 Histopathology: NR Prog: no resp to medical Tx surg Tx was postponed to aft COVID‐19 pandemic
Day. et al. (2020)^27^	52‐year‐old female	Hx: generalized ache and pain, several Fx in different healing stages, wheelchair‐bound due to weakness Ph/Ex: Severe generalized weakness RAD: Brain MRI, anteroinferior falcine meningioma, inner table dura meningioma contacting the falx in the calvarial vertex, Gadolinium PET: Both tumors had somatostatin avidity Lab: Declined Ph, Increased Alkp, PTH is an elevated, significant increase in FGF23 of serum and urinary phosphate wasting, NL Ca and 25(OH)VitD. Histopathology: not done	Tx: Surgery was high‐risk, and the patient declined. Phosphate repletion was unsuccessful, and finally, the patient was approached by burosumab, an antiFGF‐23 monoclonal antibody, at a dose of 70 mg per month. After 7 weeks, the patient's clinical and laboratory condition improved significantly, and he was able to stand.
Hudairi. et al. (2022)^18^	61‐year‐old female	Hx: DM and multiple unprovoked Fx, Ph/Ex: Severe generalized weakness and pain sensitive to touch RAD: Octreotide scan: hot lesion on Left upper thigh, Lab: Declined Ph, 25(OH)VitD, Increased Alkp, remarkable increase in FGF23 of serum and urinary phosphate wasting, NL Ca.	Tx: Surgical excision + empiric Tx with Ph and Ca Histopathology: bland spindle cell proliferation/ hemangiopericytomatous‐like blood vessels, multinucleated giant cells, smudgy/grungy basophilic matrix, and microcystic changes with scarce mitosis FU: The patient was stable after the surgery and discharged asymptomatic w/o any sign of recurrence in his 6‐month FU
Huang. Et al., (2022)^28^	51‐year‐old male	Hx: progressive pain in his feet from 2 years ago and pain in feet, hips, knees, ribs, waist, and shoulders Ph/Ex: Severe generalized weakness and pain sensitive to touch RAD: CT and MRI showed several instances of insufficient Fx. PET: tumor‐derived hypophosphate osteomalacia of the right iliac wing Lab: Declined Ph, Increased Alkp, remarkable increase in FGF23 of serum and urinary phosphate wasting, NL Ca and 25(OH)VitD	Tx: surgery was substantially effective, and after 9 months, the symptoms were resolved, and the lab data also became NL Histopathology: Infiltrative cell growth was seen. Abundant uniform oval and short spindle‐shaped cells distributed around the blood vessel in a sheet and band, rich in blood vessels interstitium, hemangiopericytoma‐like blood vessels, bone‐like matrix

Abbreviations: AbNL, abnormal; Alk, alkaline phosphatase; BMD, bone mineral density; BM, bone marrow; Ca, calcium; CT, computed tomography scan; DEXA, dual‐energy X‐ray absorptiometry; Dx, Diagnosis; DM, diabetes mellitus; Fx, fracture; FU, follow‐up; GH, growth hormone; Hx, History; IGF‐1, Insuline‐like growth hormone 1; LAB, laboratory, Lt, Left; Ext, external; LBP, low back pain; MRI, magnetic resonance imaging; NL, NormalOP, osteoporosis, NSAID, non‐steroid anti‐inflammatory drugs; NR, not‐reported; Ph, phosphate; RAD, radiology; Rt, Right; Tx, Treatment; Vx, Vertebrae; VitD, Vitamin D; Y, year; WBBC, whole body bone Scintigraphy; W/O, without.

## AUTHOR CONTRIBUTIONS

**Mahdieh Fatollahzadeh:** Conceptualization; formal analysis; methodology; project administration; software; supervision; visualization; writing – original draft; writing – review and editing. **Hamid Reza Aghaei Meybodi:** Conceptualization; formal analysis; methodology; project administration; software; supervision; visualization; writing – original draft; writing – review and editing. **Hamid Pajavand:** Formal analysis; investigation; methodology; software. **Moloud Payab:** Formal analysis; investigation; methodology; software. **Mahbube Ebrahimpur:** Conceptualization; data curation; writing – original draft; writing – review and editing. **Pouya Ebrahimi:** Data curation; writing – original draft; writing – review and editing.

## FUNDING INFORMATION

No funds have been received for this study.

## CONFLICT OF INTEREST STATEMENT

Authors declared no conflict of interest.

## CONSENT

Written informed consent was obtained from the patient for this study based on the patient's consent journal's policy.

## Data Availability

Further data will be provided by the corresponding author in case of reasonable request.
